# A Review on Grammatical Gender Agreement in Speech Production

**DOI:** 10.3389/fpsyg.2018.02754

**Published:** 2019-01-14

**Authors:** Man Wang, Niels O. Schiller

**Affiliations:** ^1^School of Foreign Languages, Qingdao University, Qingdao, China; ^2^Leiden University Center for Linguistics, Leiden, Netherlands; ^3^Leiden Institute for Brain and Cognition, Leiden, Netherlands

**Keywords:** grammatical gender, agreement, lexico-syntactic feature, speech production, ERP

## Abstract

Grammatical gender agreement has been well addressed in language comprehension but less so in language production. The present article discusses the arguments derived from the most prominent language production models on the representation and processing of the grammatical gender of nouns in language production and then reviews recent empirical studies that provide some answers to these arguments.

## Introduction

In order to successfully convey a message when speaking, speakers need to encode the to-be-produced speech in a grammatically correct way. Language systems differ in terms of whether or not grammatical gender is distinguished in the systems. Some language systems do not distinguish the grammatical gender of nouns, such as English and Chinese. Some other language systems (e.g., Romance languages, German, Dutch, and Russian, but also many non-Indo-European languages) distinguish nouns according to their grammatical gender (e.g., masculine versus feminine, common versus neutral). Very often, the grammatical gender of the nouns bears an opaque relation to the biological gender of its referent (i.e., the conceptual or natural gender; see Schiller and Caramazza, 2003; Schwichtenberg and Schiller, 2004).

Grammatical gender agreement is a crucial part of syntactic agreement within a noun phrase and within a sentence (e.g., in Spanish: ‘La rosa es roja.’ The_fem_ rose_fem_ is red_fem_). It is stored in the mental lexicon as a lexico-syntactic feature of words (see Levelt et al., 1999a; Nickels et al., 2015). Unlike the feature ‘number,’ which always needs to be activated based on the concept (e.g., ‘one cat’ or ‘two cats’; see Schiller and Caramazza, 2002) and requires the selection of the *–s* suffix in English for regular plural nouns (Nickels et al., 2015), ‘gender’ is an intrinsic feature of nouns (Corbett, 1991). Its activation has little to do with the concept and does not always have morphological or phonological consequences. For instance, in Romance languages such as Italian and Spanish, nouns’ suffixes are morphologically and phonologically marked by the grammatical gender, although the gender-to-ending correspondence is not always transparent (see, e.g., Padovani et al., 2005).

Psycholinguistic models of language production have made distinctive assumptions about the representation and processing of grammatical gender in speech production. For instance, the WEAVER++ model distinguishes a conceptual stratum, a syntactic stratum and a word-form stratum (Levelt, 1992; Roelofs, 1992, 1993; Roelofs and Meyer, 1998; Levelt et al., 1999a,b) and words are linked to their syntactic features (i.e., grammatical gender, grammatical class, and number) at the syntactic stratum (see Levelt et al., 1999a; Nickels et al., 2015). This model distinguishes between the activation and selection of the syntactic features. Specifically, the grammatical gender is only selected when it is needed for production (Roelofs, 1992, 1993). The WEAVER++ model assumes the seriality of processing stages and a unidirectional link from a word to its syntactic features (see also, Jescheniak and Levelt, 1994). By contrast, although constructed with the same layered architecture, the ‘interactive’ spreading-activation model (Dell, 1986, 1988, 1990; Dell and O’Seaghdha, 1991, 1992) assumes an interactive manner of activation flow. In other words, the links between layers are bi-directional. Alternatively, the ‘Independent-Network’ model (Caramazza, 1997; Caramazza and Miozzo, 1997) assumes three independent networks: the lexical-semantic network, the syntactic network and the phonological lexemes. In this latter model, the lexical-semantic network can directly activate the syntactic network and the phonological lexemes in parallel. Please note that both Dell’s interactive model and the Independent-Network model reject the seriality and discreteness of activation flow and in principle allow the bypassing of the retrieval of grammatical gender to specify the phonological form of noun phrases when the grammatical gender of the nouns is not explicitly marked in their phonological forms (see Schriefers and Jescheniak, 1999 for a discussion).

There have been heated debates over the underlying mechanism of the selection of freestanding and bound gender-marking morphemes in speech production (see Jescheniak et al., 2014 for a thorough review). Jescheniak et al. (2014) reviewed empirical evidence and concluded that both gender-marked freestanding morphemes like determiners and bound morphemes like adjectival inflections are selected by competition at the phonological level in speech production (but see, Schiller and Costa, 2006). Compared to the review by Jescheniak et al. (2014), which focuses on the gender-marked morphemes, our review focuses on the activation and selection of the abstract gender features of the noun during speech production. Two major questions arise from the assumptions of the three most prominent language production models. The first one is whether or not grammatical gender is automatically activated and selected in speech production even when it is not explicitly needed for speech production. The second one is whether grammatical gender can be bypassed when the phonological form can be generated without knowing its gender. We will discuss empirical evidence on these arguments.

Empirical studies have collected evidence from speech errors as well as error-free speech. Studies that analyze speech errors give hints on the representation and processing of grammatical gender in speech production (see Schriefers and Jescheniak, 1999 for a thorough review). For example, German noun substitution errors show that the intended and intruded nouns were often of the same gender and this phenomenon occurs even without syntactic cues, consistent with a two-stage language production model (Marx, 1999). Evidence from Tip-of-the-Tongue (TOT) errors demonstrates that speakers can access to grammatical gender when no phonological cues are available, suggesting separate representations of lexico-syntactic features and phonological forms (Vigliocco et al., 1997; but see also Caramazza and Miozzo, 1997; Miozzo and Caramazza, 1997 in Italian; Gonzalez and Miralles, 1997 in Spanish; cf. Schriefers and Jescheniak, 1999, p. 589). Furthermore, studies of anomia and TOT states in Italian, Spanish, French and German where a noun is usually produced in a full NP (e.g., with a gender-marking determiner) show that patients have gender knowledge when they fail to name (e.g., Badecker et al., 1995; Vigliocco et al., 1996; Marx, 1999; cf. Friedmann and Biran, 2003). By contrast, Hebrew-speaking aphasic patients do not preserve grammatical gender in bare noun naming (Friedmann and Biran, 2003). However, for most speech error studies that investigate the grammatical gender representation and processing in language production, the results fail to give clear conclusions on lexical access in language production under error-free circumstances (for naturally occurring speech errors, see, e.g., Barbaud et al., 1982; Berg, 1992; Vigliocco et al., 1997; for experimentally elicited speech errors, see, Meyer and Bock, 1999; Vigliocco and Franck, 1999; see Schriefers and Jescheniak, 1999 for a detailed review on studies analyzing speech errors). Therefore, this article will focus on discussing studies that analyze error-free speech.

## Behavioral Studies

Experimental studies have made use of the picture-word interference paradigm (PWI) to investigate the processing of syntactic features in speech production. The PWI paradigm (e.g., Glaser, 1992; see MacLeod, 1991 for a review) has been widely used to examine the language production process. Schriefers (1993) presented colored pictures to participants while a distractor word whose grammatical gender was either congruent or incongruent with that of the target picture was superimposed on the picture. Participants were asked to name the target pictures using noun phrases while ignoring the distractors. The experiment was conducted in Dutch with native Dutch speakers. In Dutch, there are two grammatical gender categories: neutral and common gender. The results of the study showed that participants were faster in naming the pictures when the grammatical gender of the distractor word (e.g., ‘dak,’ roof_neuter_) was congruent with that of the target picture name (e.g., ‘boek,’ book_neuter_) than an incongruent condition with a distractor (e.g., ‘tafel,’ table_common_). This was also true with both article-adjective-noun (e.g., ‘het groene boek,’ the green book) and plain adjective-noun (e.g., ‘groene boek,’ green book) productions. The difference in naming latencies was called ‘the gender congruency effect’ and this effect was also observed in definite article-noun production (e.g., ‘de tafel,’ the table) in Dutch (van Berkum, 1997; La Heij et al., 1998; Schiller and Caramazza, 2003; Starreveld and La Heij, 2004; Schiller, 2013), in noun phrase naming in German (Schriefers and Teruel, 2000; Schiller and Caramazza, 2003), Chinese (Wang et al., 2006; Zhang and Liu, 2009), Konso (Tsegaye, 2017; Tsegaye et al., unpublished), Croatian (Costa et al., 2003), and Czech (Bordag and Pechmann, 2008).

Schriefers (1993) claimed that the target word’s grammatical gender feature (e.g., neuter) and the distractor’s gender feature (e.g., common) compete for selection when they are incongruent. The competition in the selection of the word’s grammatical gender causes interference when producing the target noun phrase. This account has been called the *gender selection interference hypothesis* (GSIH; Schiller and Caramazza, 2003, 2006). This hypothesis assumes the selection of the grammatical gender. Another study by Schriefers and Teruel (1999) on French noun phrase production also showed the gender congruency effect even when the definite article and the post-nominal adjective were identical for nouns of different grammatical genders (e.g., ‘l’assiette jaune,’ the_fem or masc_ dish_fem_ yellow_fem or masc_) (but see Experiments 2 and 3 in Bordag and Pechmann, 2008). These findings, especially the latter one, suggest that the selection of grammatical gender cannot be bypassed, which runs against the interactive model and the Independent-Network model, both of which in principle allow such a bypass.

Nevertheless, conflicts have been found in later studies in various languages. The so-called gender congruency effect was not replicated in Italian definite article-noun phrase production (Miozzo and Caramazza, 1999; Cubelli et al., 2005) when the article is determined by both the grammatical gender (masculine versus feminine) and the phonological form (e.g., the onset) of the noun. Similar results were observed in other studies in Italian (Miozzo et al., 2002) and other Romance languages, such as Spanish, Catalan (Costa et al., 1999) and French (Alario and Caramazza, 2002; see Caramazza et al., 2001 for a review). Miozzo and Caramazza (1999) attributed the discrepancy to cross-linguistic differences in the selection of determiners. In Dutch, the selection of determiners depends on the noun’s gender and number features, whereas the determiner selection in Italian also depends on the phonological form of the subsequent word. Furthermore, Schiller and Caramazza (2003) asked German and Dutch speakers to name pictures using “determiner and/or adjective” single or plural noun phrases. In German and Dutch, determiners are identical if the nouns are in plural forms. The so-called gender congruency effect was only obtained when to-be-named pictures were in singular forms, not in plural forms when the determiner was identical for all genders. The gender congruency effect was then interpreted as reflecting the competition in the selection of determiner forms, i.e., the *determiner selection interference hypothesis* (DSIH) (see also Schiller and Caramazza, 2006). These findings suggest that the selection of grammatical gender can be bypassed if its information is not necessary to determine the phonological form of the to-be-produced speech (see Jescheniak et al., 2014 for a detailed review over the selection of gender-marked morphemes in speech production). However, these results do not answer directly whether or not the grammatical gender feature is automatically activated when it does not have any phonological consequences.

Discrepancies were also observed in bare noun naming. No gender or determiner congruency effect was observed in bare noun naming in Dutch (La Heij et al., 1998; Starreveld and La Heij, 2004). In a Greek (L1) to German (L2) translation task, the gender congruency effect was only observed in noun phrases when the target utterance required gender agreement (Salamoura and Williams, 2007), although gender information in L2 is assumed to be computed anew during production rather than stored as a fixed feature in L1 (Bordag and Pechmann, 2007). By contrast, Cubelli et al. (2005) observed the grammatical gender interference effect in Italian bare noun production even when grammatical gender is not necessary for producing the target (but see also Finocchiaro et al., 2011). The gender congruency effect in bare noun naming was also found in Konso in a study by Tsegaye et al. (2013), Tsegaye (2017), Tsegaye et al. (unpublished) and in Czech where the congruency effect was shown with a comparable feature, i.e., declensional class (Bordag and Pechmann, 2009). Cubelli et al. (2005) concluded that the grammatical gender is selected even in bare noun production. The grammatical gender effect was observed both when the gender-to-ending correspondence is transparent (i.e., *-a* for feminine and *-o* for masculine) and when it is opaque (i.e., *-e* for either feminine or masculine). Paolieri et al. (2010, 2011) replicated this effect in both Italian and another Romance language, Spanish, which has an analogous gender system. Paolieri et al. (2011) extended the previous finding in that differential effects were observed when the morphological transparency of the ending vowel for gender varied. For instance, for the target word ‘trattore’ (tractor_masc_), the gender congruency effect was stronger when the distractors had the same ending *-e* (e.g., ‘peperone,’ pepper_masc_ vs. ‘cicatrice,’ scar_fem_) in contrast to different endings (e.g., ‘cappello,’ hat_masc_ vs. ‘batteria,’ drums_fem_). Emerging evidence shows that in Romance languages such as Italian and Spanish, the selection of grammatical gender is not bypassed and the grammatical gender effect is related to the gender-to-ending transparency (Paolieri et al., 2011).

It seems that grammatical gender plays a crucial role in accessing the phonological form of the noun which may contribute to the selection of grammatical gender in bare noun production in Romance languages. Cubelli et al. (2005) proposed a Double Selection model, in which a word’s lemma is linked to a semantic category node and a grammatical gender node in a two-layered structure. In spoken word production, both the lexico-semantic representation and the lexico-syntactic representation have to be selected prior to accessing the phonological form at the second layer. According to Cubelli et al. (2005), the discrepancy between the findings in Dutch and Italian bare noun productions is attributed to language-specific properties. The compulsory selection of grammatical gender is only present in languages with a complex morphological structure such as Italian, and can be bypassed in languages with a relatively simple morphological structure such as Dutch.

The Double Selection model proposed by Cubelli et al. (2005) is in line with the WEAVER++ model in that the grammatical gender information is accessed prior to the word’s phonological form. Nevertheless, it disagrees with the WEAVER++ model by assuming a direct link between the semantic representation and the phonological representation. This, however, is in line with the prediction of the IN model and allows the bypass of grammatical gender selection in bare noun production as observed in Dutch. Furthermore, in contrast to the prediction of the IN model, the Double Selection model assumes the compulsory competition in the selection of grammatical gender as reflected by the grammatical gender effect in Italian bare noun production. This does not fully contradict the conjecture of the WEAVER++ model which assumes that the grammatical gender feature is activated but not selected if it is not needed for production (Roelofs, 1992, 1993) since the Double Selection model restricts the compulsory selection of the grammatical gender information to languages with a complex morphological structure. Nevertheless, whether the grammatical gender feature is automatically activated or not is still open to debate. Unfortunately, the existing behavioral data cannot provide evidence for resolving this debate.

## Electrophysiological Studies

In contrast with behavioral data, such as naming latencies which only reflect the outcome of the speech production process, electrophysiological data can provide fine-grained measurements of online processing of the speech production process (Luck, 2005). However, electrophysiological studies investigating the grammatical gender processing in language production are scarce. Van Turennout et al. (1998) measured the Lateralized Readiness Potentials (LRPs) in two versions of a combined forced choice task and go/no-go task and showed that the retrieval of grammatical gender feature precedes the retrieval of the phonological form information. Another study by Barber and Carreiras (2005) showed that grammatical gender disagreement elicited an N400 effect in (silent) sentence reading in Spanish.

In order to test whether lexico-syntactic features are activated and selected in bare noun production, Wang et al. (2018) investigated the Chinese language, where grammatical gender is not marked but nouns have a comparable lexico-syntactic feature, i.e., classifiers. It is compulsory to use a classifier between an article, a quantifier or another modifier and its associated noun (e.g., ‘yi1 pi3 ma3,’^1^ one *classifier-pi3* horse). Chinese classifiers bare a transparent semantic relationship to the noun but opaque in other cases (Tzeng et al., 1991) and are considered to have some functions of determiners in other languages (Cheng and Sybesma, 2005). Using the PWI paradigm, the authors asked participants to name the target picture in bare nouns with a distractor that was either classifier-congruent or -incongruent with that of a target picture. A stronger N400 effect was observed on the classifier-incongruent trials compared to the congruent trials (both semantically unrelated), suggesting the automatic activation of classifier feature in bare noun production. By contrast, no effect in naming latencies was observed between the classifier-congruent and -incongruent conditions, suggesting that the classifier feature is not selected in the process of bare noun production when it is not needed. The bypass of the selection of classifier feature is compatible with the hypothesis by Cubelli et al. (2005) given that Chinese has a very simple morphological structure. The findings are also compatible with the assumption by the WEAVER++ model that the lexico-syntactic feature is automatically activated but not selected in language production when it’s not needed (Roelofs, 1992, 1993). Nevertheless, it is yet unclear to what extent these findings can be generalized to other language systems, especially those that distinguish the grammatical gender of nouns.

A few studies have investigated the processing of grammatical gender agreement in sentence comprehension. Molinaro et al. (2011) reviewed nine studies examining the neural correlates of either determiner-noun or noun-adjective gender mismatches. It has been observed that the N2pc component was modulated in a grammatical gender agreement task in Italian word pairs whose gender is transparently marked (Caffarra et al., 2013). The involvement of gender-to-ending is also shown in the investigation of language comprehension. Gender-to-ending transparency is shown to modulate grammatical gender effect in the gender categorization task. Specifically, the gender congruency effect was observed in morphologically complex words and even in pseudo-morphological words but not in nouns without morpheme-like parts (Meunier et al., 2008). In the following discussion on neural imaging evidence, it is suggested that language perception and production share a common neural network for grammatical gender processing (Heim et al., 2002; Miceli et al., 2002).

## Functional Magnetic Resonance Imaging (fMRI) Studies

Alongside the ongoing debate about whether grammatical gender is selected even when it is not needed for production investigated mainly with the behavioral measurements, researchers also investigated the neural correlates of grammatical gender retrieval using fMRI. Distinctive neural mechanisms seem to underlie the processing of the grammatical gender at different levels (Heim et al., 2002). While syntactic processing at the sentence level involves pronounced activation in the inferior part of Broca’s area (e.g., Friederici et al., 2000; Indefrey et al., 2001), the selection of grammatical gender is correlated with the activation in the superior part of the Broca’s area when participants were producing determiners (Heim et al., 2002; more specifically Brodmann’s Area, (BA) 44, see Heim et al., 2009) or identifying the grammatical gender of a given word (Miceli et al., 2002). The superior part of Broca’s area is found to be activated in both comprehension and production tasks, suggesting a common neural network for grammatical gender processing in language perception and production (Heim et al., 2002; Miceli et al., 2002).

Although the activation associated with accessing the grammatical gender information is located in the Broca’s area – i.e., BA 44/45 – the focus of the activation varies depending on participants’ processing strategies (Heim et al., 2005). Specifically, the direct access to gender information when performing the gender judgment features a network involving the inferior tip of BA 44. Alternatively, when participants adopt an indirect, form-related strategy, i.e., producing the definite determiner in order to judge the grammatical gender of the given word, they demonstrate a network of activation in BA 45/47, the superior part of BA 44 and the fronto-median wall (Heim et al., 2002, 2005, 2009; Miceli et al., 2002). The distinctive foci of networks were in line with a dual-route model for the retrieval of grammatical gender proposed by Gollan and Frost (2001) based on their behavioral study, with one route of direct grammatical gender access and the other being more form-based. Gollan and Frost (2001) also pointed out that the cross-linguistic variability in grammatical gender-marking may lead to variance in the speed and availability of the form-based route to grammatical gender. The influence of gender-marking regularity is confirmed by another fMRI study, showing activation in the left and right fronto-temporal areas (Padovani et al., 2005). By varying the gender-to-ending regularity of Italian words, the authors observed a complex activation network and suggested a lexically based route for words with “opaque” and “irregular” gender-to-ending correspondences and a form-based route for “transparent” words.

Emerging evidence suggests the importance of gender-to-ending regularity and the transparency of gender-marking. Furthermore, the distinctive routes of grammatical gender retrieval may result from the variability within and across languages in these two factors.

## Summary

The empirical studies discussed in the present article have investigated the representation and processing of grammatical gender or a similar lexico-syntactic feature in language production. It is generally agreed that grammatical gender is represented as a separate lexico-syntactic feature in the mental lexicon.

However, several issues still remain unsolved concerning the processing of grammatical gender in language production. Firstly, it seems that grammatical gender is not selected in bare noun production when it is not necessary for production in Dutch and Chinese but is selected in Italian and Konso (Tsegaye et al., 2013; Tsegaye, 2017; Tsegaye et al., unpublished) bare noun production. Further evidence is needed to confirm Cubelli et al. (2005)’s argument that the discrepancy is attributed to the complexity of morphological structure of the target language. Using another language other than Italian and Konso that has a complex morphological structure would illuminate this matter. Secondly, the study in Chinese provides evidence for the automatic activation of the lexico-syntactic feature, i.e., classifier, in bare noun production. To our knowledge, no direct evidence has been drawn to test whether it is the same with the grammatical gender feature. Thirdly, few studies have looked into the manner of activation flow between a word and its syntactic feature to determine when and how the lexico-syntactic feature is activated in language production.

Furthermore, it is still open to debate whether the selection of grammatical gender is bypassed in noun phrase production when the selection of grammatical gender does not have any phonological consequence. Nevertheless, emerging evidence has shown distinctive mechanisms underlying the selection of grammatical gender in Romance languages like Italian and Spanish, and Germanic languages like German and Dutch. For instance, the grammatical gender congruency effect in bare noun production was observed in Italian but not in German or Dutch; the determiner congruency effect was observed in German and Dutch but not in Romance languages (but see Schriefers and Teruel, 1999). fMRI studies also provide evidence for distinctive neural networks for the processing of grammatical gender and suggest that participants tend to adopt a more form-related route to access gender information in Romance languages where the gender-to-ending regularity modulates the gender effect. By contrast, participants tend to adopt a more lexically based route to access grammatical gender in Dutch and German where the noun’s morpho-phonological form is generally not strongly marked by gender.

In sum, the present article reviewed recent empirical studies on the representation and processing of grammatical gender of nouns in language production. We may not have exhausted all relevant studies but the empirical evidence discussed above will provide reference in constructing the language production model.

## Author Contributions

Both authors have contributed equally to the manuscript and approved it for publication.

## Conflict of Interest Statement

The authors declare that the research was conducted in the absence of any commercial or financial relationships that could be construed as a potential conflict of interest.

## Footnotes

1 As an example, “yi” indicates the phonetic notation of the lexical item, i.e., Pinyin of the word and the number 1 indicates the Lexical Tone 1.
